# Supplementary material for: Retinol saturase coordinates liver metabolism by regulating ChREBP activity
Source: Nat Commun. 2017 Aug 30;8:384. doi: 10.1038/s41467-017-00430-w (PMC5577314; doi:10.1038/s41467-017-00430-w)
Supplement: Supplementary file 1 — Supplementary Information [file 41467_2017_430_MOESM1_ESM.pdf]

Description of Supplementary Files

File name: Supplementary Information
Description: Supplementary figures and supplementary tables.

File name: Peer review file

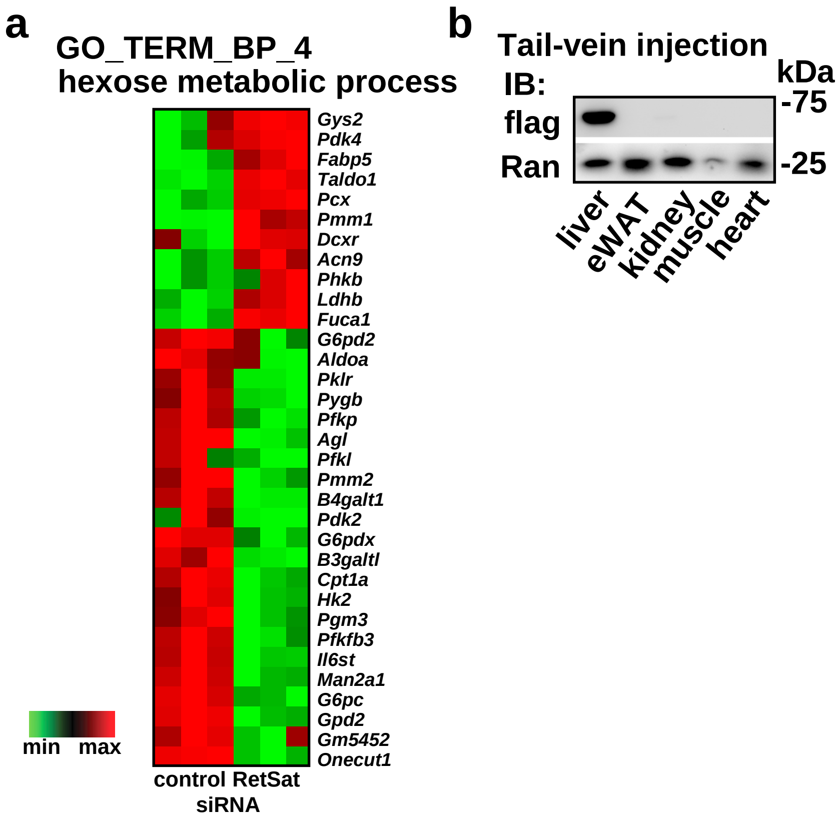

Supplementary Figure 1 I RetSat depletion alters the expression of genes involved in glucose metabolism and liver specificity of adenoviral expression in mice. (**a**) An expression heatmap of regulated genes mapped to GO_TERM_BP_4 'hexose metabolic process' by RetSat depletion in primary hepatocytes. (**b**) An adenovirus expressing a flag-tagged protein was injected via the tail-vein and protein expression in the indicated organs was determined 72 h later by immunoblotting.

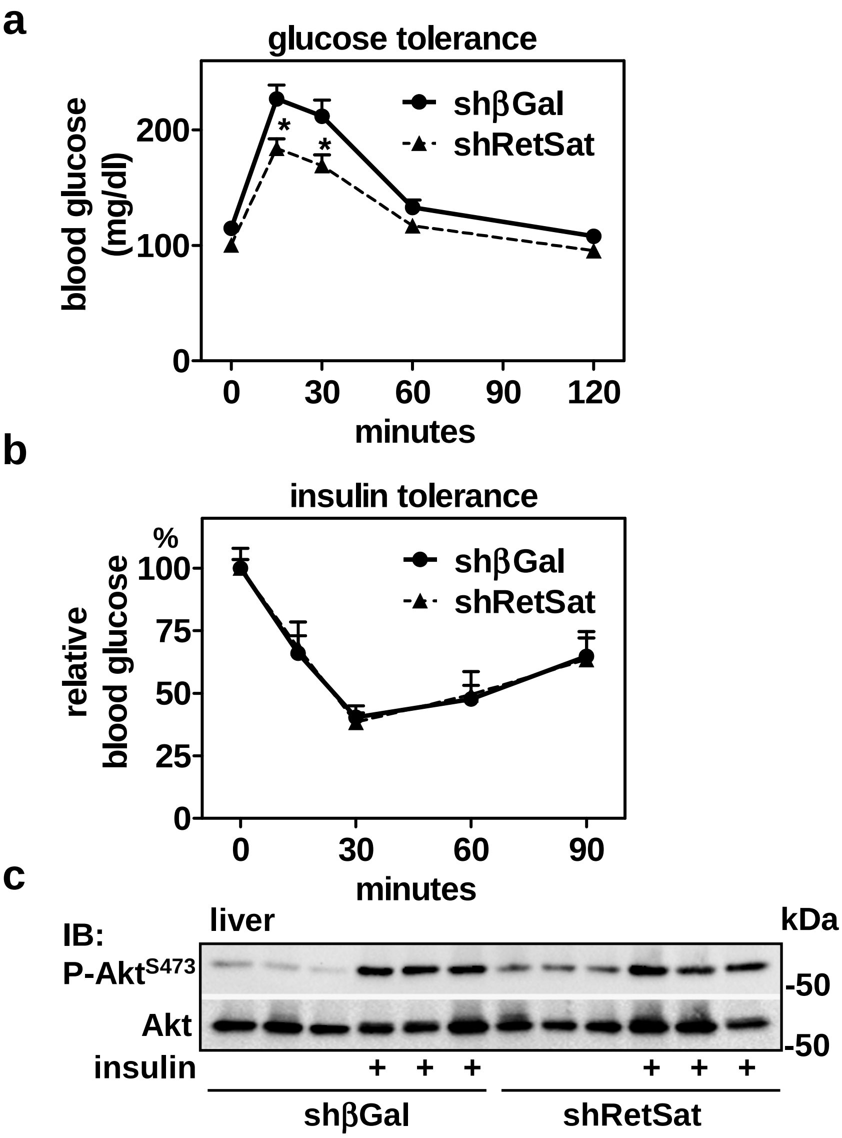

Supplementary Figure 2 I Glucose and insulin tolerance of mice depleted of hepatic RetSat. Mice fed HS/HFD were depleted of hepatic RetSat by adenoviral expression of shβGal or shRetSat and (**a**) glucose and (**b**) insulin tolerance determined. Data are shown as mean ± s.e.m., n=8 (shβGal), 6 (shRetSat); **P*<0.05 between shβGal und shRetSat by two-way RM ANOVA with Bonferroni post test. (**c**) Mice fed HS/HFD were depleted of hepatic RetSat by adenoviral expression of shβGal or shRetSat and injected intraperitoneally with 0.9% saline with or without 1U/kg insulin. 10 min later, mice were euthanized and hepatic expression of the indicated proteins determined.

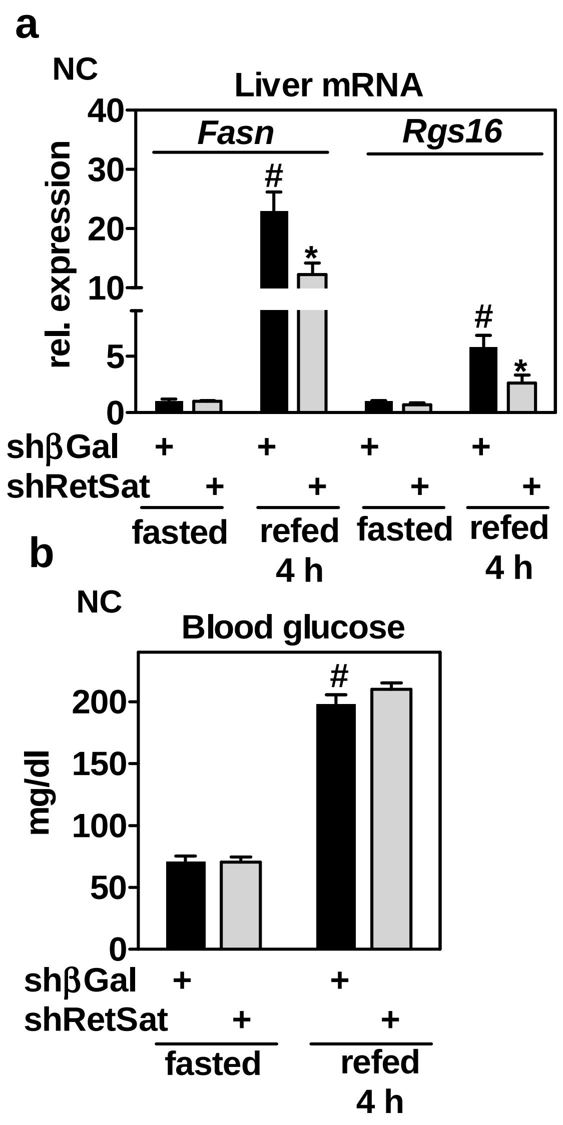

Supplementary Figure 3 I RetSat depletion in mice fed NC impairs the induction of ChREBP target genes upon re-feeding, independently of blood glucose levels (**a**) Mice fed NC were injected with adenoviruses expressing siRNA targeting βGal or RetSat. 5 days later, mice were fasted for 24 h and re-fed with NC for 4 h. mRNA expression of *Fasn* and *Rgs16* was determined by qPCR. (**b**) Fasted and re-fed blood glucose levels of mice described in (***a***). Data are shown as mean ± s.e.m of n=5 (fasted shβGal), 5 (fasted shRetSat), 8 (refed shβGal), 7 (refed shRetSat); two-way ANOVA with Bonferroni post test showed significances between fasted and refed mice ([#]*P*<0.05) and between shβGal and shRetSat (**P*<0.05) as indicated.

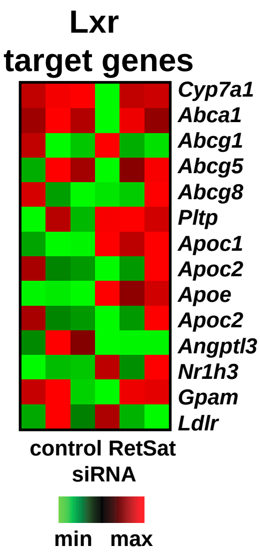

Supplementary Figure 4 I Effects of RetSat depletion on Lxrα target gene expression. An expression heatmap of selected Lxrα target genes in primary hepatocytes with or without RetSat depletion.

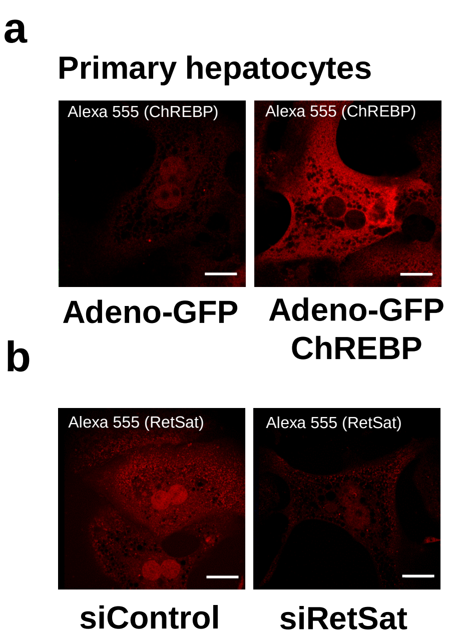

Supplementary Figure 5 I Validation of ChREBP antibody and RetSat depletion. (**a**) Primary hepatocytes were seeded on glass cover slips and treated with adenoviruses expressing GFP or a GFP-ChREBP fusion for 48 h. After fixation, ChREBP was stained by immunocytochemistry and analyzed by confocal microscopy. (**b**) Primary hepatocytes were seeded on cover slips and treated with Control or RetSat siRNA for 48 h. After fixation, endogenous RetSat was stained by immunohistochemistry and analyzed by confocal microscopy, scale bars = 20 µM.

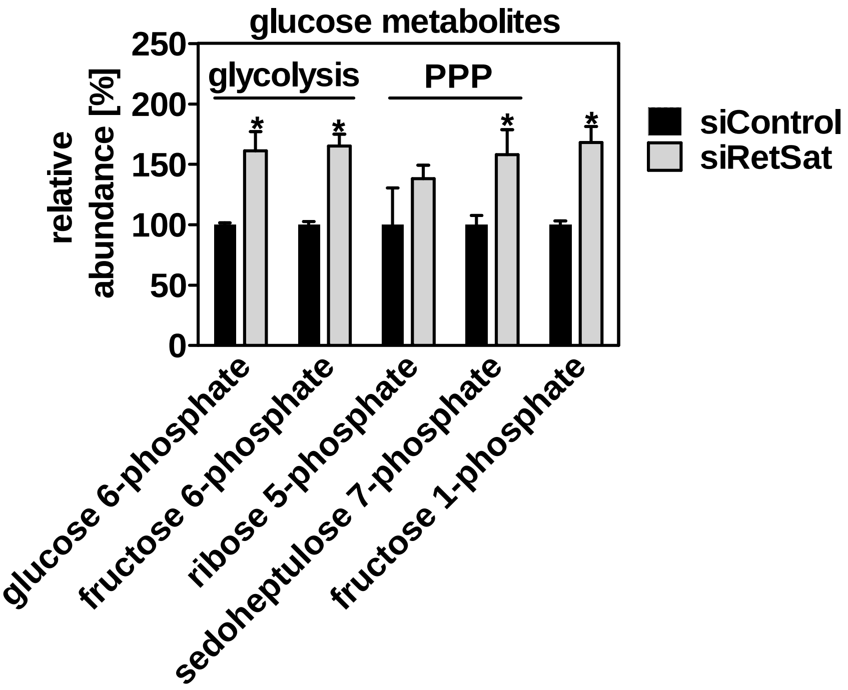

Supplementary Figure 6 I RetSat depletion in primary hepatocytes leads to an accumulation of glucose metabolites. Relative abundance of glucose metabolites in siControl or siRetSat treated hepatocytes was assessed by mass spectroscopy. Data are shown as mean ± s.d., n=4 independent transfections of hepatocyte cultures from the same mouse; *$P<0.05$ by two-tailed *t* test.

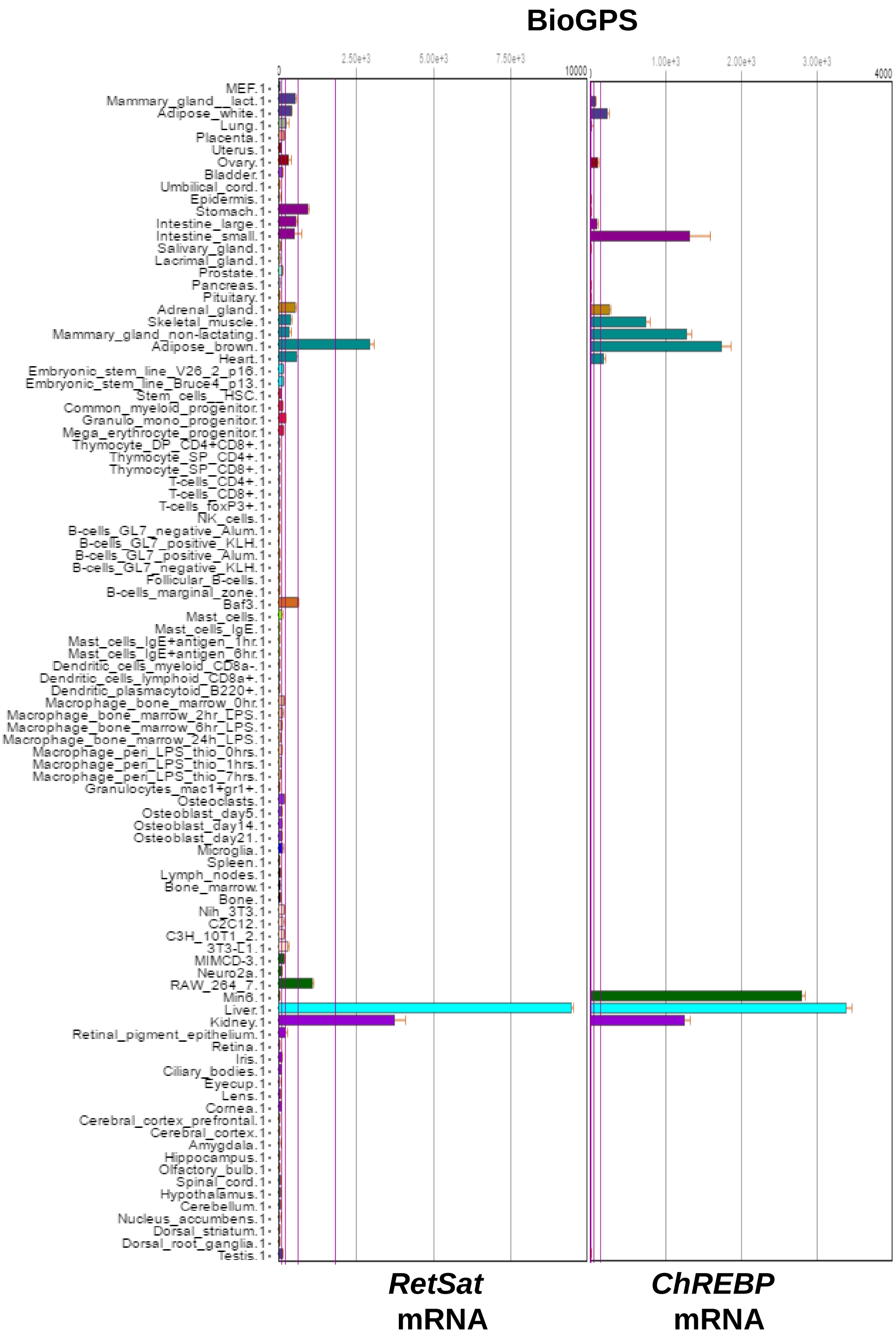

Supplementary Figure 7 I Comparison mRNA expression profile of *RetSat* and *ChREBP*. Gene expression profiles of murine *RetSat* and *ChREBP* from BioGPS (probesets 1424715_at and 1419185_a_at, respectively).

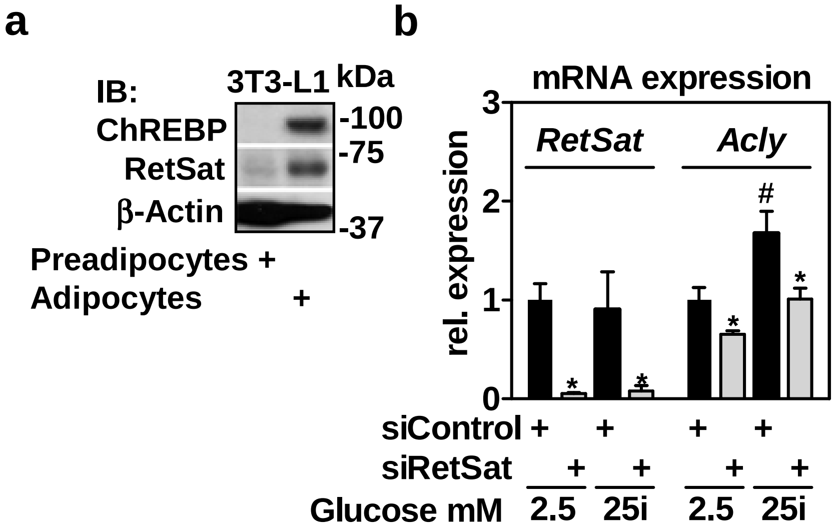

Supplementary Figure 8 I Expression of RetSat and ChREBP proteins in 3T3-L1 adipocytes and impaired glucose sensing in adipocytes depleted of RetSat. (**a**) RetSat and CHREBP protein expression in undifferentiated and differentiated 3T3-L1 adipocytes was determined by immunoblotting. (**b**) 3T3-L1 were electroporated with control or RetSat siRNA and 24 h later exposed to either low or high glucose concentrations and 100 nM insulin as indicated. After an additional 24 h, mRNA expression of *RetSat* and *Acly* was determined by qPCR. Data are shown as mean ± s.d., n=6 independent transfections of 3T3-L1 adipocytes; two-way ANOVA with Bonferroni post test revealed significances between low and high glucose concentrations (#*P*<0.05) and between siControl and siRetSat (**P*<0.05).

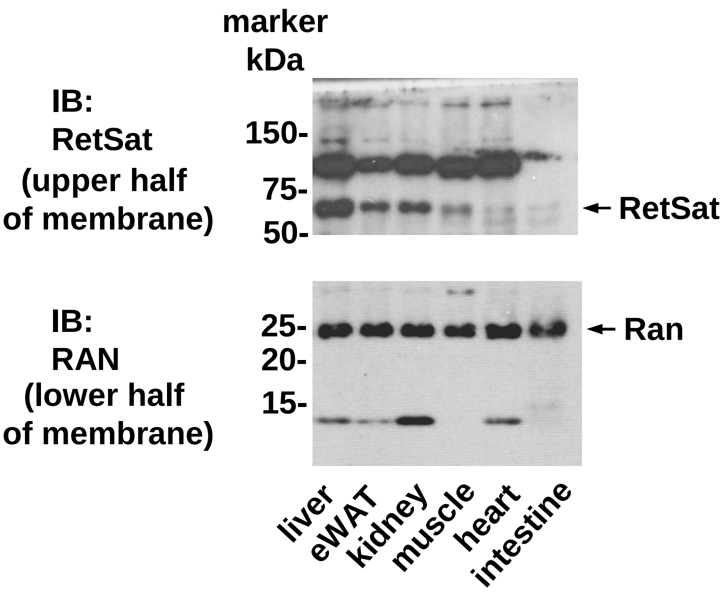

Supplementary Figure 9 I Immunoblot scan of Fig. 1a

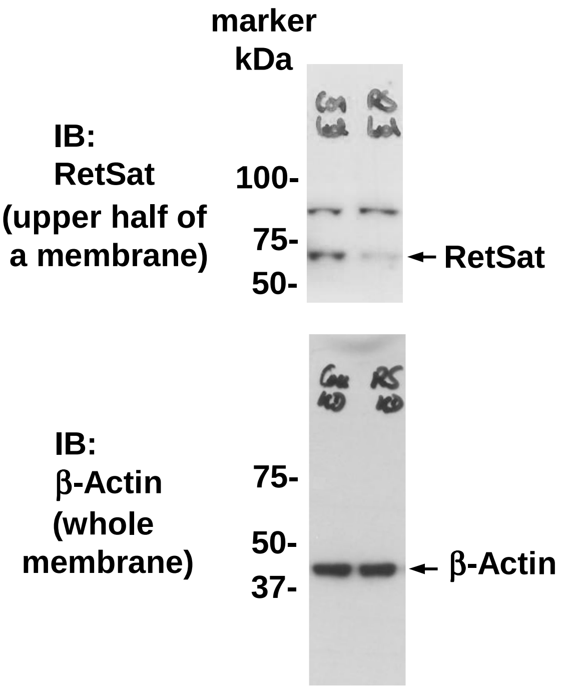

Supplementary Figure 10 I Immunoblot scan of Fig. 1b

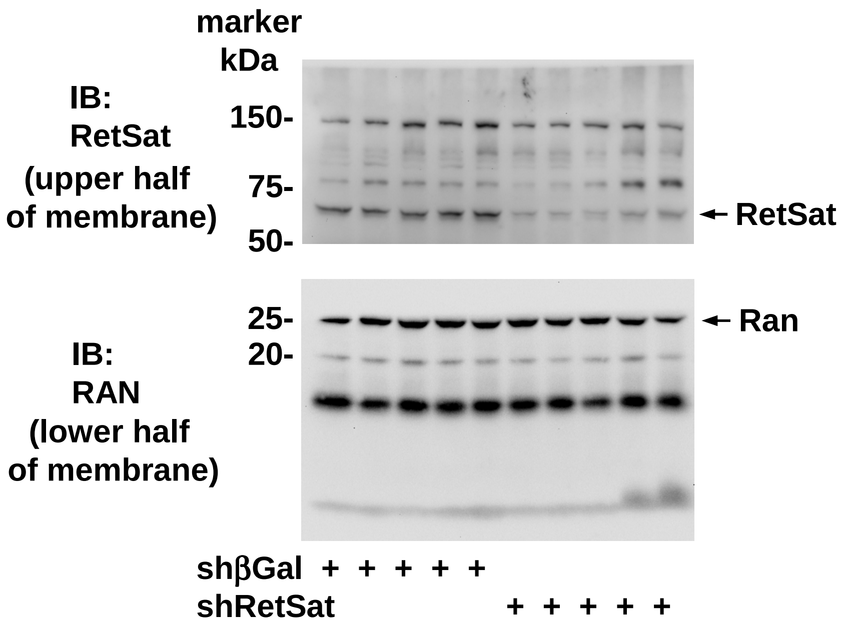

Supplementary Figure 11 I Immunoblot scan of Fig. 2b

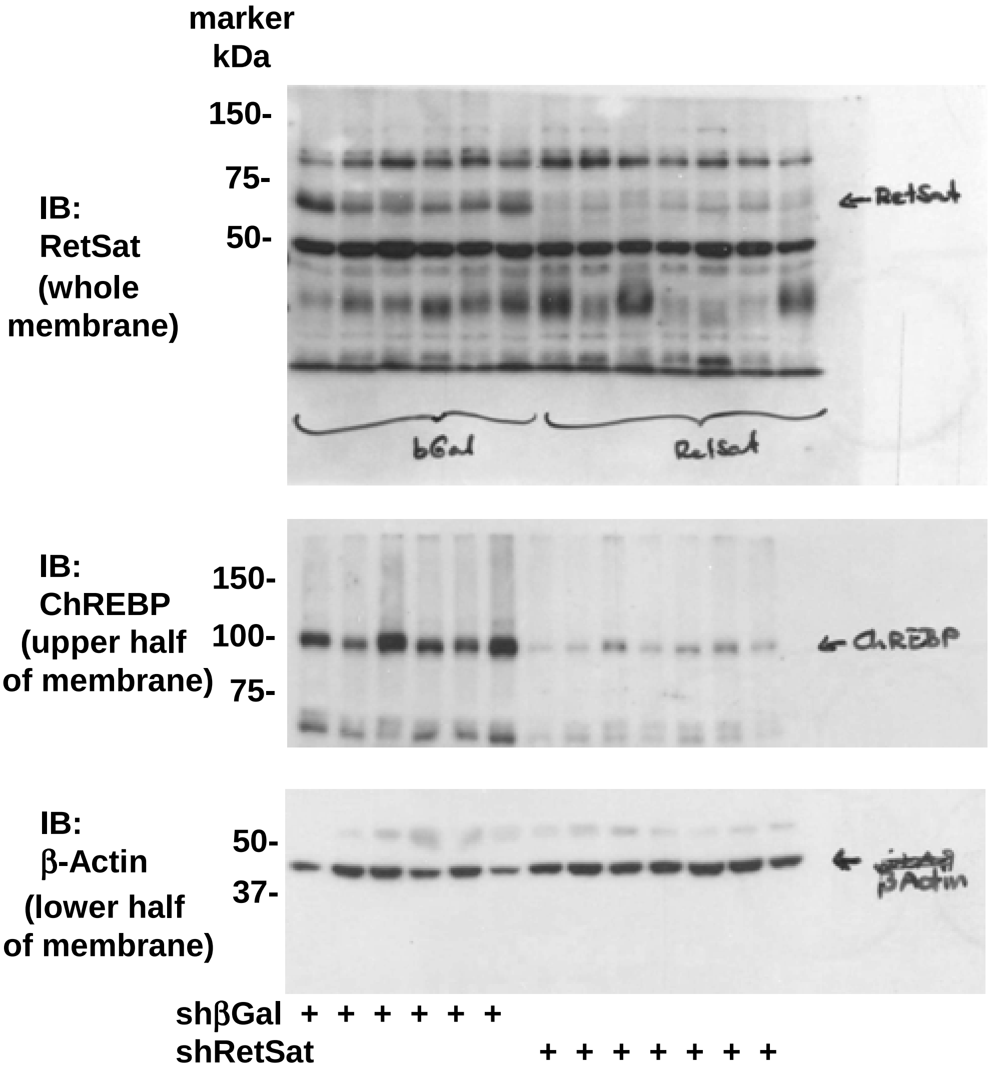

Supplementary Figure 12 I Immunoblot scan of Fig. 4b

Supplementary Table 1.

Metabolic parameters of NC-fed shβGal and shRetSat mice (mean ± s.e.m.)

	**shβGal (n=13)**	**shRetSat (n=12)**
ad libitum blood glucose (mg/dl)	166.85±4.32	173.45±8.76
fasted blood glucose (mg/dl)	70.85 ±4.60	70.5 ±5.14
ad libitum insulin (ng/ml)	0.87±0.15	0.47±0.18
ad libitum serum TGs (mg/dl)	176±9.33	184±15.51
ad libitum serum NEFA (mM)	1.92±0.06	1.92±0.09

Supplementary Table 2.

Human subject characteristics (mean ± s.e.m.)

n (male)	29 (12)
age (years)	60±3
BMI (kg/m^2)	27.1±1.2
waist circumference (cm)	98±3
SBP (mm Hg)	137±4
DBP (mm Hg)	73±3
ALT (U/L)	40.6±5.9
NAFLD activity score (0-8)	1.6±0.3
Liver steatosis (%)	14.5±3.1

Supplementary Table 3.

Oligonucleotide sequences used in this study (5'-3')

**qPCR**	m36B4 fw	TCATCCAGCAGGTGTTTGACA
	m36B4 rv	GGCACCGAGGCAACAGTT
	mAcc1 fw	GGTGAAGCTGGACCTAGAAGAGAA
	mAcc1 rv	AAAGGCCAAACCATCCTGTAAGC
	mAcly fw	GATCTGGACCATGGTTGCT
	mAcly rv	CCGTAATTCGCCAGTTCATT
	mAdipor2 fw	GGGTCTCCCGACTCTTCTCT
	mAdipor2 rv	GAAGCAAGGTTGTGGGTTACA
	mBhlhe40 fw	CAGCTGAAGGATCTCCTACCC
	mBhlhe40 rv	TCAATGCTTTCACGTGCTTC
	mChREBPtotal fw	CAGCCCAGCCTAGATGACTT
	mChREBPtotal rv	CAAAGCTGGGGGACTCTATG
	mChREBPα fw	CGACACTCACCCACCTCTTC
	mChREBPα rv	TTGTTCAGCCGGATCTTGTC
	mChREBPβ fw	TCTGCAGATCGCGTGGAG
	mChREBPβ rv	CTTGTCCCGGCATAGCAAC
	mDgat1 fw	GATCTGAGGTGCCATCGTCT
	mDgat1 rv	GGATCAGCATCACCACACAC
	mElovl6 fw	AAAGCACCCGAACTAGGTGA
	mElovl6 rv	AGGAGCACAGTGATGTGGTG
	mFasn fw	CACCAATACAGATGGCAGCA
	mFasn rv	CCAGCTGGCTGATACAGAGA
	mG6pc fw	CGAGGAAAGAAAAAGCCAAC
	mG6pc rv	GGGACAGACAGACGTTCAGC
	mGck fw	CTTCCCTGTAAGGCACGAAG
	mGck rv	AAGTCCCACGATGTTGTTCC
	mLxra fw	CTGCCCAGCAACAGTGTAAC
	mLxra rv	CTGAGGGTCGGGTGCAAT
	mMid1ip1 fw	GGTGAACAACATGGACCAGA
	mMid1ip1 rv	CGCTGACCTCGTCTATCTCC
	mPfkl fw	CATATATGTGGGGGCCAAAG
	mPfkl fw	CATATATGTGGGGGCCAAAG
	mPfkl rv	AGTTGGCTGGCTTGATGTTC
	mPfkl rv	AGTTGGCTGGCTTGATGTTC
	mRetSat fw	CCCATCAAGCAAGGATCCAA
	mRetSat rv	ATGGGTACCAGCGCAGTCA
	mRgs16 fw	TGGGCCAGTAAGCATAACAA
	mRgs16 rv	TTCAGCAGCAAATCGAAAGA
	mScd1 fw	CCGGGAGAATATCCTGGTTT
	mScd1 rv	GCGGTACTCACTGGCAGAGT
	mSrebp1c fw	GCGCTACCGGTCTTCTATCA
	mSrebp1c rv	TGCGCAAGACAGCAGATTTA
	mTpi fw	CTGGATCCCAAAATTGCTGT
	mTpi rv	ACCCAGGTGGCTCCTAAGTC
	mTxnip fw	GGTCTCAGCAGTGCAAACAG
	mTxnip rv	GGCCTCATGATCACCATCTC

	hACC1 fw	ACCACCTACGGATAGACCGC
	hACC1 rv	TCGCTTTGGGGAAATAAAGTG
	hHPRT fw	TGACACTGGCAAAACAATGCA
	hHPRT rv	GGTCCTTTTCACCAGCAAGCT
	hPKLR fw	GCCTACTGGACATTGACTCCGA
	hPKLR rv	TCATCCCGGCCTTGATCAT
	hRetSat fw1	CAGGACTGTTCAACACCTATGAACA
	hRetSat rv1	CCCCAGTTGCTGCTTCACA
	hRetSat fw2	GGATGTGGTGGTAATTGGCAGT
	hRetSat rv2	CCCCTGCCTTGGTATGTTGTT
**siRNA (ds)**	Control siRNA	UAG-CGA-CUA-AAC-ACA-UCA-AUU
	Retsat siRNA-1	UCA-GCC-GAG-UAC-CAG-AGA-AUU
	Retsat siRNA-2	GCU-CAA-AGG-UCA-AGG-CAC-AUU
**shRNA target sequence**	βGal	CTACACAAATCAGCGATTT
	RetSat	TCAGCCGAGTACCAGAGAA
